# A systematic synthesis of qualitative studies on parents’ experiences of participating in early intervention programs with their infant born preterm

**DOI:** 10.3389/fpsyg.2023.1172578

**Published:** 2023-07-13

**Authors:** Gunn Kristin Øberg, Marit Sørvoll, Cathrine Labori, Gay L. Girolami, Ragnhild B. Håkstad

**Affiliations:** ^1^Department of Health and Care Sciences, Faculty of Health Sciences, UiT the Arctic University of Norway, Tromsø, Norway; ^2^Department of Clinical Therapeutic Services, University Hospital North Norway, Tromsø, Norway; ^3^Department of Physical Therapy, College of Applied Health Sciences, University of Illinois at Chicago, Chicago, IL, United States

**Keywords:** early intervention, parental involvement, infants born preterm, interaction, embodiment

## Abstract

Early intervention programs involving both the parent and the infant born preterm have demonstrated positive effects on developmental outcomes for the children. However, studies have also shown that parental engagement and adherence when implementing intervention programs can be challenging. The aim of this review was to provide a comprehensive description and new insights into key messages gleaned from the parent reports on participating in early intervention with their infant born preterm; knowledge vital to facilitate implementation of early interventions into clinical practice when using a model of direct parent involvement. Early intervention is broadly defined as a multi-interdisciplinary field provided to children from birth to five years of age to foster child health, wellbeing, development, adapting parenting and family function. For this systematic synthesis we define early intervention as programs with specific activities completed with the infant during the first year after birth. We assembled qualitative interview studies on parents’ experiences with participation in early intervention and applied Malterud’s qualitative systematic meta-synthesis to synthesize and translate the original findings across studies. In the analysis we applied enactive concepts of embodiment, autonomy, participatory sensemaking, and agency. 10 qualitative studies were identified and included. The systematic synthesis reveals how parents’ successful and meaningful participation in early intervention programs were facilitated by their “active embodied doing.” The “embodied doing” appeared as the basis for the parents’ sense-making processes, development of confidence, and the ability for parents to see new possibilities for actions within themselves, with and in the child. In that respect, a perception of mutuality in the interaction between parent, infant and interventionist was central. Consequently, an important consideration when implementing early intervention into clinical practice is to promote embodied parent–infant interactions as well as trust between the parent and the interventionist.

## 1. Introduction

Infants born preterm can have long term challenges concerning their social, cognitive, and motor development. Challenges are related to behavioral and emotional problems (e.g., temperament, hyperactivity, phobias, and self-concept), attentional deficits, several cognitive functions (e.g., intelligence, memory, language, and visuospatial processing), and increased risk for learning problems (e.g., mathematics and reading attainments) (Wolke, 1998; Johnson et al., 2016; Robinson et al., 2022; Trickett et al., 2022). A risk of various motor impairments (e.g., delayed motor development, coordination problems, and cerebral palsy) are also present. The incidence of serious neurological and intellectual sequelae increases with decreasing gestational age and birth weight (Johnson et al., 2016; Trickett et al., 2022). Experience and active participation are suggested to enhance neurodevelopment through structural and functional reorganization of the brain (Merzenich et al., 2014; Mottahedin et al., 2017), implying that experience and participation may alter neurodevelopment in infants born preterm through experience-expectant plasticity (Knickmeyer et al., 2008; Spittle and Treyvaud, 2016). Various early intervention programs have been designed and implemented with the goal of enhancing early neurodevelopment and minimizing the potential for long-term impairments associated with preterm birth. Early intervention include health care, education, and social care professionals and is generally defined as a multi-interdisciplinary service provided to children from birth to five years of age (Shonkoff and Meisels, 2000). For this paper, we define early intervention as programs with specific activities completed with the infant during the first year after birth. In this context some early intervention programs are provided solely in the neonatal intensive care unit (NICU) (Als et al., 2012; Øberg et al., 2012), others commence in the NICU and go beyond discharge from the hospital (Landsem, 2016), while others are offered solely after discharge (Hilderman and Harris, 2014; Spittle et al., 2015; Cooper et al., 2020). Study outcomes indicate the most beneficial programs for cognitive and motor outcomes are those that continue or commence post hospital discharge (Spittle et al., 2015) as well as those involving both the infant and the parent (Vanderveen et al., 2009; Spittle et al., 2015; Hughes et al., 2016; Spittle and Treyvaud, 2016).

As part of early intervention programs, parents often receive training and/or guidance on how to support their child to promote the infant’s well-being and neurodevelopment as well as adaptive parenting (Benzies et al., 2013; Hughes et al., 2016; Landsem, 2016; Spittle and Treyvaud, 2016). The latter seem to be of great importance as the research indicates responsive interactions between parents and infants born preterm are critical for optimal child development (Landsem, 2016; Spittle and Treyvaud, 2016; Anderson et al., 2020). However, the research also highlights there is a significant risk for parental psychological distress after giving birth prematurely, which might make participation in the intervention protocols difficult and/or stressful (Dusing et al., 2008; Lutz et al., 2009; Ionio et al., 2019). Indeed, parents who experience depression, anxiety, or stress, may have difficulty interacting with their young infant (Anderson et al., 2020; Cheong et al., 2020) creating implications for their ability to participate in early intervention programs.

Parents frequently report increased care-giving burdens after giving birth prematurely as well as lower self-confidence in caring for their preterm infant (Treyvaud, 2014; Hughes et al., 2016; Spittle and Treyvaud, 2016). Although the research indicates parents of infants born preterm value participating in interventions to promote their child’s development (Lutz et al., 2009; Øberg et al., 2019), parents have also described active participation as stressful and overwhelming (Dusing et al., 2008; Harniess et al., 2022). Furthermore, studies also show that compliance in parent participation in intervention programs can be challenging (Kynø et al., 2012; Lillo-Navarro et al., 2015).

Therefore, to engender enthusiasm for and adherence to intervention programs, it appears critical that health care professionals working with parents of preterm infants appreciate how early parent administered interventions may affect the parent. The integration of findings from a range of qualitative studies within the field can help identify factors important to the families; those which should be considered when involving parents in early interventions. However, we could find no meta-analysis providing a holistic view of the results of qualitative studies, nor a robust account of the meaning and the significance of those results as they pertain to parents. Therefore, we designed this study to explore and synthesize qualitative research published in peer reviewed journals describing parents’ experiences with involvement in early intervention programs with their infant born preterm. The aim of this study was to go beyond summaries of results from primary qualitative studies and reconceptualize the findings to offer a more comprehensive description and new insights into what parents of infants born preterm report as meaningful and motivating when participating in early intervention programs. We also hoped to uncover a deeper understanding of the key messages gleaned from the parent reports.

## 2. Methods

Qualitative research methods offer an opportunity to access and understand the world of peoples’ experience and meaning, their practices and the relationships they imply and create (Thornquist, 2018). Therefore, exploration of parents’ personal thoughts, attitudes, reflections, and bodily experiences of participating in early intervention programs with their infants born preterm, may promote understanding and optimization of professional practice with families. In this article, a systematic qualitative review based on the guidelines of Malterud’s qualitative meta-synthesis (Malterud, 2019) was conducted. Two of the authors (CL, RBH) independently completed a computerized literature search in four databases which included Web of Science, Medline, Embase and CINAHL. We limited our search to qualitative studies published in peer reviewed journals as most scientific data are published in this form. Before 1995 it was rare to include parents of infants born preterm in early interventions. The literature search therefore targeted articles published in peer review journals 1995–2021. With authors that are native English and Scandinavian speaking, we filtered for English and Scandinavian language publications. The search took place in October and November 2021 and was updated in September 2022. The research strategy comprised the following terms and keywords: preterm OR premature OR risk AND interview^*^ AND parent^*^ AND experience^*^ OR perception^*^ OR relation^*^ OR bonding OR attitude^*^ OR sensitivity OR attachment OR competence^*^ AND intervention OR treatment OR therapy OR program AND infant.

**Figure 1:**
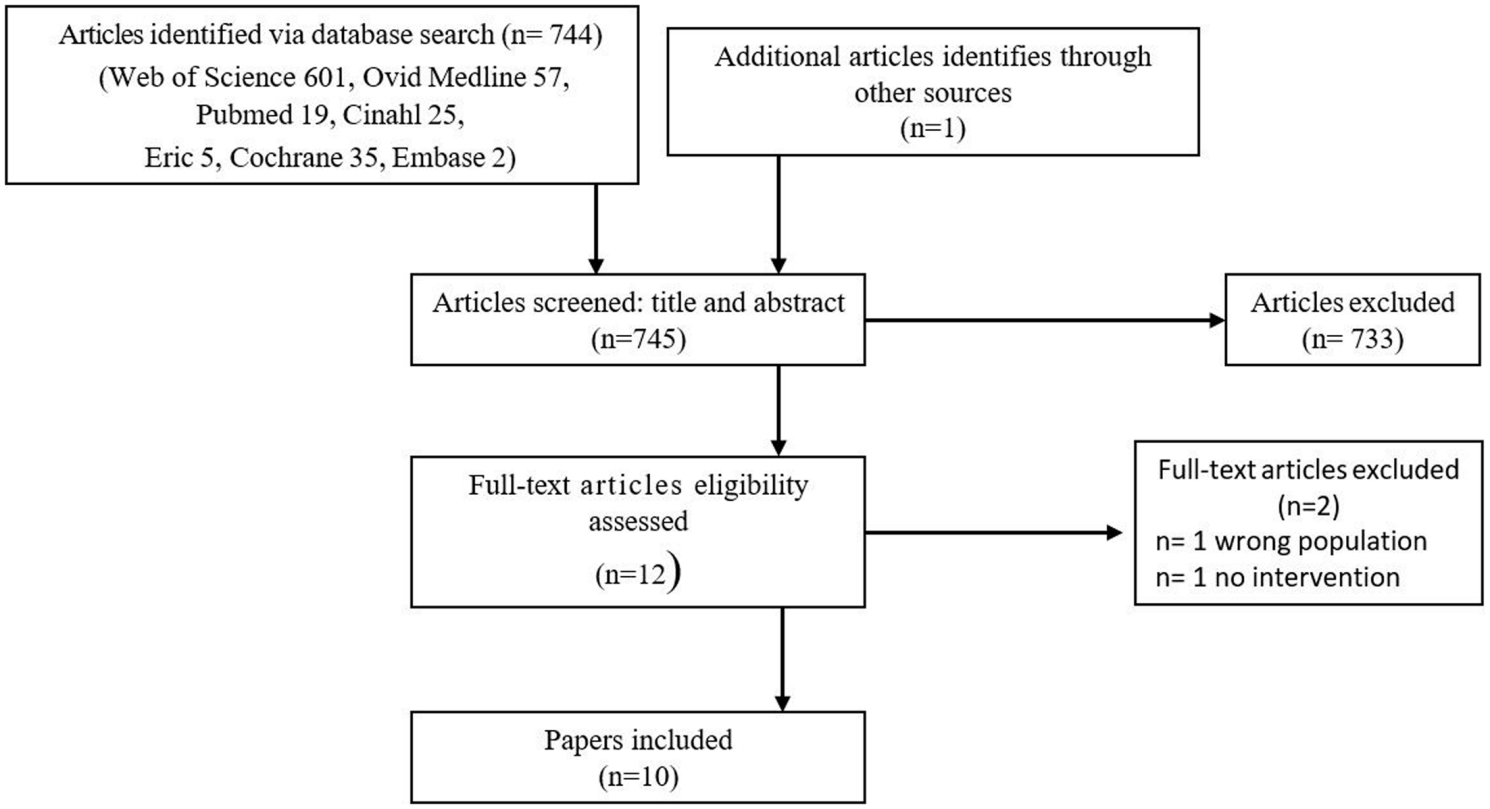
Literature search flow diagram.

The titles and abstracts of the articles were selected for further review based on these inclusion criteria: (1) the interviewees were mothers or fathers of infants born preterm (gestational age <37 weeks at birth), (2) the parents and their infant had completed an early intervention program that commenced before six month corrected age (CA), (3) the early intervention included a motor developmental goal, (4) a qualitative interview design was used, and (5) the articles were original research, published in peer-reviewed scientific journals. Articles were excluded if questionnaires were used and if the early intervention program solely focused on behavioral-, cognitive-, and social development or a mix of these developmental areas, i.e., motor development was not addressed within the scope of the study. When title and abstract did not provide sufficient information, the full text was retrieved. No additional articles were identified from the reference lists of the relevant articles. However, one article (Edney and Mchugh, 2021) was identified through a recommendation at ResearchGate. No new articles were added after the update in September 2022. The total hits were 745. The results of the literature search are presented in Figure 1.

The methodological quality of 11 studies was evaluated based on the guidelines accompanying the “Critical Review Form-Qualitative Studies (Version 2.0).” The evaluation included the study purpose, relevant background literature, methods and design, theoretical framework, sampling and data collection, data analysis, findings, conclusions, and implications. The form, provided by McMaster University (Letts et al., 2007), was independently completed by two of the authors (either CL-RBH, or CL-GKØ, or CL-MS, or RBH-GKØ, or MS-GKØ). Any disagreements were determined by consensus among at least three of the authors. After performing the critical review on the 11 articles, one article was excluded because it did not meet our inclusion criteria (Gibbs et al., 2019). The remaining 10 articles were accepted as having sufficient quality for further analysis. As the rate was consistent with recommendations for qualitative meta-synthesis (Chrastina, 2018), we considered the total inclusion as sufficient. One study was based on data from a focus group interview (Kynø et al., 2013), another on a semi-structured telephone interview (Edney and Mchugh, 2021), while the rest were individual qualitative semi-structured interviews (Gravem et al., 2013; Benzies and Magill-Evans, 2015; Håkstad et al., 2016; Lee et al., 2019; Øberg et al., 2019; Baraldi et al., 2020; Van Den Hoogen et al., 2021; Ochandorena-Acha et al., 2022). A total of 193 parents of infants born preterm, from eight different countries, were represented in these articles. Further details regarding the study setting and the early intervention programs are provided in Table 1.

**Table 1:** Summary of results.

Study	Design	Participants	Location	Interventionist	Intervention characteristics	Findings
Baraldi et al. (2020) (Sweden)	Semi-structured interviews	17 parents of 14 children born extremely preterm	HOME	Psychologist, pediatrician, and therapists (physiotherapist, occupational therapist, speech- and language therapist, dietician)	Stockholm Preterm Interaction-Based Intervention (SPIBI): home visits, focusing on parent–child interaction, understanding children’s cues and encouraging the next developmental step.	Intervention as a sense of security
						Knowledgeable interventionists being able to find suitable tips and advice
Benzies and Magill-Evans (2015) (Canada)	Semi-structured interviews	85 fathers of late-preterm infants	HOME	Female home visitors	Video-modeled play intervention aiming to teach sensitivity and responsiveness to infant cues.	Importance of interaction.
						Validation of the parental role. Confidence in the parental role
						Positive with tailored feedback.
Edney and McHugh (2021) (United Kingdom)	Semi-structured telephone interviews	9 mothers of high-risk infants (prematurity 55%, genetic conditions 22%, congenital heart defects 11%, brain injury 11%)	NICU	Physiotherapist, occupational therapist, speech- and language therapist	Therapy services when infant was referred to therapy complementing developmental care and family-integrated care practices that routinely take place on the NICU	Participation in therapy as a means of regaining autonomy and control.
						Barriers related to accessing therapists and external demands on their time.
Gravem et al. (2013) (United States)	Semi-structured interviews	10 mothers of infants born preterm	NICU and HOME	Nurses, occupational therapists, other health care providers	Assisted early exercises following discharge	Exercises as beneficial for the infants
						Increased bonding
						Feeling “scared” of hurting the infants during the first few days of home exercise.
						Further teaching and learning alleviate the fears
Håkstad et al. (2016) (Norway)	Semi-structured interviews	9 parents of 7 infants born preterm (including one set of twins)	HOME and PT location	Physiotherapist	Physiotherapy: Embodied interaction and exploration of child capacity	Learning about their child’s individuality give confidence necessary to support and care for their child in everyday life. Progression toward normalization.
Kynø et al. (2013) (Norway)	Semi-structured focus group interviews	14 parents of 11 infants born preterm	NICU and HOME	Nurse	Mother-Infant Transaction Program (MITP): focuses on both child development and parent–infant relationship. The aim of MITP was to teach the parent how to be sensitive and responsive to their infant’s physiological, behavioral, and social cues to enhance child development.	Reducing parental stress and enhancing confidence in the parental role. Increased competence and secure caring for their preterm born child.
						Program perceived as educational and supportive.
Lee et al. (2019) (ROK)	In-depth interviews	12 mothers of Low-birth-weight infants	HOME	Different service providers, determined by experts.	A follow-up program; structured home visit, professional education, self-help group meetings and individualized counseling for acceleration of child development. Learning and performing physical stimulation and body games.	Increase of mothers’ confidence in their parenting skills. Becoming sensitive to the child’s needs.
						Increased mother’ attachment.
Ochandorena-Acha et al. (2022) (Spain)	Open interviews and Semi-structured interviews	15 mothers and fathers of 14 infants born preterm	NICU and HOME	Physiotherapist	Early physiotherapy intervention: a preventative approach aimed to enhance the parent–infant relationship, parents’ knowledge about infants’ cues and management strategies, and improve motor development.	Increased parental competence, empowered to take care of the infant, and enhancement of infant’s development.
						Capable of developing coping strategies.
Van Den Hoogen et al. (2021) (Netherlands)	11 Semi-structured interviews	13 parents of 11 infants born preterm (9 mothers and two couples of fathers and mothers)	NICU	Interdisciplinary (nurses and other health care professions)	The VOICE (values, opportunities, integration, control, evaluation) aiming to engage parents in developmental care, to decrease stress, and to increase empowerment	Parents felt strengthened and were empowered in their role as primary caretakers.
Øberg et al. (2019) (Norway)	11 Semi-structured interviews	6 parents of 7 infants (one pair of twins)	NICU	Physiotherapist	Functional movement intervention program	Reinforced attachment between the parent and the child. Parent–infant bonding and parental empowerment and competency.

The process of data extraction from the 10 included studies was completed by three of the authors (RBH, CL, and GKØ). The authors closely read the results sections in the articles, first individually, followed by a collaborative discussion process to reflect on, and identify preliminary concepts adequate for the research question of this study. Two preliminary concepts were identified: (1) early intervention as a means for strengthened parent–child relationship and (2) building parent empowerment through mutual interactions with the child.

The next step in the synthesis and analysis was to establish how the 10 studies were related. The procedure, comparing relevant text extracts from the included primary studies, is called translation (Chrastina, 2018; Malterud, 2019). Translation was accomplished by searching for empirical findings representing each of the two preliminary concepts. Text extracts from the 10 articles, referring to each of the preliminary overarching concepts, were randomly organized. Studies were listed horizontally and findings of relevance vertically. The study by Øberg et al. (2019) contributed rich data to both concepts. In addition, the article’s systematic presentation of the results, made it the best article to designate as the index study. The next step was to sort related findings from the nine remaining articles into the same horizontal rows. As recommended by Malterud (2019), we processed text extracts within each row, condensed, and synthesized them into common categories. During this interpretation process, we explored the categories for conflicting findings across the included studies. The two preliminary concepts were subdivided into two and three categories, respectively. Finally, the final conceptualization of the two preliminary concepts was translated to patterns comprising relational aspects and parental empowerment (Tables 2, 3).

**Table 2:** Worksheet: matrix for synthesis and analysis regarding relationship pattern.

Øberg et al. (2019)	Gravem et al. (2013)	Kynø et al. (2013)	Benzies and Magill-Evans (2015)	Håkstad et al. (2016)	Lee et al. (2019)	Baraldi et al. (2020)	Ochandorena-Acha et al. (2022)	Van Den Hoogen et al. (2021)	Edney and McHugh (2021)	Our translation	Relationship pattern
Feeling a closeness they had not experienced before.	Intervention fostered increased bonding with their babies		Being recognized by the baby.		Gained an under- standing of attachment.			“To hold him and to care for him gave me warm feelings and contributed to stronger feelings of being a mother.”		Taking an active part and spending time in mutual interactions gave a new closeness and stronger parental feelings	Active learning and doing embodied interactive interventions build mutuality in the parent–child relationship
Mutuality in interaction had a substantial impact on bonding.	The exercise intervention was additional time that they may not otherwise have spent with their infants		The fathers liked “the guidance for interactions between dad and baby,” “reading the baby’s body language,” and “to see myself and how I interact and how she responds.”		It became much more natural to play with their babies.						
Reinforced attachment between the parent and child.					Bodily play made the mother feel closer to the baby						
We learned about each other during the interaction.					They tried to get close with their babies by playing body games						
It was very nice to be allowed to interact with my own baby, not just sitting with her in my lap with all those tubes as my only experience… she became more like MY BABY	Developed increased responsiveness.	Learned about the child’s capacity to maintain interaction	More aware of being responsive to the baby.	Better understand the child’s capabilities.	Learned to become sensitive to their babies’ expressions and needs.	Trust in infant’s capacity.		PT/OT gave knowledge on infant behavior and handling.		Parents get to know, interact, and challenge their child through learning about the infant’s bodily expressions, capacity, and development.	
A turning point in their interactions with and perceptions about their infant.	Parents adjusted exercise program to infant state and responses		Seeing things that you do not usually think about.	Involvement in therapy is where they get to challenge the child, not just nurse	Watched carefully how the child changes.	Learning to recognize what the child understands gave parent an “aha-moment”		Parent-to-parent exchanges gave new knowledge about infant behavior			
Observing the infant’s independent movements validated the baby’s competence.											

**Table 3:** Worksheet: matrix for synthesis and analysis regarding parental empowerment pattern.

Øberg et al. (2019)	Gravem et al. (2013)	Kynø et al. (2013)	Benzies and Magill-Evans (2015)	Håkstad et al. (2016)	Lee et al. (2019)	Baraldi et al. (2020)	Ochandorena-Acha et al. (2022)	van den Hoogen et al. (2021)	Edney and McHugh (2021)	OUR TRANSLATION	EMPOWERMENT PATTERN
Administrating the intervention gave a liberating feeling and marked a turning point - “the baby became mine”	To hold and play with your baby is enjoyable	Learning about the infant’s state and accessibility in interaction gives confidence in parental role.	Affirmation of their role as a father. Reflect on various aspects of their role. Fathers learned to play with their child	Therapeutic assignments gave a touch of normalcy	Started to be hopeful that they could become good parents.	Participating in intervention brought a sense of security.	Empowered when caring for their baby and promoting their development.	Active participation empowered the parental role and made them feel complete as a parent.	Involvement in therapies as a means to regain control over their child’s destiny and learn how to help their child develop.	Active participation and learning are key to confidence and fulfillment in the parental role	Building knowledge through interactions with their child together with a skillful therapist fades fear and enables parents to fulfill their role with a sense of autonomy, confidence and enthusiasm
	Enlightening to get to know my baby’s body and see him develop.			Intervention became integrated in everyday life activities together with the infant	Felt more comfortable about parenting.	Home visits give perspective regarding child’s development.	More capable of handling their baby.	Learned by watching and copying health care practitioner	Valued and found relevance in developmental therapies.		
				Learning to support motor development gave reassurance and confidence.			Parents felt more able to stimulate and play with the child		Participation was a natural part of parenting and gave confidence		
									Parents were confident and satisfied that they had positively influenced infant development		
No longer fearful of moving their babies.	More prepared and more confident.	Obtaining knowledge made it easier to accept new challenges and gave a tool and security for improvising.	Feel more comfortable being alone with her.	Reassurance and confidence with respect to their handling of their children.	It became much more natural to play with their babies.	Learning to strengthen motor development brought a sense of security.	Lost the fear of harming the child.	Health care support, confirmation and compliments gave confidence	Limited access to therapist reduced parent’s sense of confidence	Gaining knowledge together with their therapist faded initial fears and promoted self-efficacy and enthusiasm	
The baby seemed more real and less fragile – not a doll made of glass	Training sessions alleviated fears and prepared parents to perform exercises in the home			Nothing is too much if it can help the baby	Learned to control problematic behaviors with maternal affection and interaction instead of staying confused or afraid.	Lack of fear because we could ask all questions.	Alleviating stressors such as feeling useless and insecure.		Valued involvement in therapy in the phase when worries about disability and excitement about milestones go hand in hand		
“It was scary when we started, but under supervision I became more experienced touching and moving my baby....it became less scary”						A knowledgeable interventionist can prevent parental concerns.			Initial concerns about hurting the infant were overcome with support from therapists		
The transition from the role of passive parent to active parent–child interaction was also challenging.									The more you know the more you can help the baby		
Heightened control in their decision-making.	Parents adjusted exercise program to infant state and responses	Tried out what they had learned and experience that it worked.	Were able to transfer their learning to other contexts.	Influence the ability to understand how to care for the child.	They learned to become sensitive to their babies’ expressions and needs, and use appropriate parenting skills	Practical part of EI was highlighted as helpful.	Learning a way to increase competency related to care of their baby.	Parents went from feeling powerless to fully participating in care and decisions.	Regained autonomy and felt more control over their infant’s outcomes and future.	The gaining of knowledge makes the parents feel competent and promotes autonomy in daily life decision-making	
I became more secure about what I could do or not do with my baby early on		Parents learned something that is beyond knowledge just about the baby, and could apply what they had learned from the intervention to a new child.					Confident to do things.	Helped parents express how they wished to participate in caring for their infant.	Limited access to therapist reduced parent’s sense of confidence		

## 3. Results

In this section we first present the two preliminary concepts representing our synthesis of the results from the included articles. Subtitles represent the categories under each theme and communicate the condensed meaning of each category. The findings are emphasized with selected quotations from the included articles. Finally, we address our translation to patterns within each of the two preliminary concepts.

### 3.1. Early intervention as a means for strengthened parent–child relationship

#### 3.1.1. Taking an active part and spending time in mutual interactions gave a new closeness to their infant and stronger parental feelings

Results from five of the primary studies offered a broad range of descriptions about the association between taking an active parental role in administrating early interventions and the development of the parent–infant relationships (Gravem et al., 2013; Benzies and Magill-Evans, 2015; Lee et al., 2019; Øberg et al., 2019; Van Den Hoogen et al., 2021). Exercises and body play were important components. Most important to the development of the parent–child relationship was what the parents learned about interactions with their infants. Core elements mentioned were learning about infant neurobehavior and how to read and act on their infants’ cues to generate sensitive and effective responsiveness (Benzies and Magill-Evans, 2015; Lee et al., 2019; Øberg et al., 2019; Van Den Hoogen et al., 2021). For some parents, just holding their infant contributed to closeness (Van Den Hoogen et al., 2021). However, most parents highlighted that to act in interaction with their infant, was what brought them significantly closer together with their child (Benzies and Magill-Evans, 2015; Lee et al., 2019; Øberg et al., 2019). One mother who learned and practiced interaction with her child through play activities stated:

“I think I have become much closer to my child, and I am surprised due to something difficult to explain. It seemed that we naturally became closer to one another as we played together without realizing that we were. I now feel more connected to my child” (Lee et al., 2019).

Through mutual interaction in which parents and children learned about each other, parents had the experience of seeing and being seen by their child (Benzies and Magill-Evans, 2015; Øberg et al., 2019). They expressed that their active part-taking in the interventions strengthened their parental role and made them prioritize and discover the value of mutual interactions with their child (Gravem et al., 2013;Benzies and Magill-Evans, 2015; Lee et al., 2019; Øberg et al., 2019). In the NICU parents felt a closeness they had not previously experienced (Øberg et al., 2019). One mother indicated this closeness enhanced her parental feeling and said:

“I think it was great to get trained to administer the intervention. It was very nice to be allowed to interact with my own baby, not just sit with her in my lap with all those tubes as my only experience… I could see that she was more than that and it was not so dangerous to move her… She was a baby. She became more like MY BABY… it was possible to touch her, right?” (Øberg et al., 2019).

In the study of fathers experiences later in infancy, the experience of being recognized by their infant was an exhilarating experience for fathers and their parental feelings (Benzies and Magill-Evans, 2015). One father said:

“I enjoy that he smiles at me, that I make him happy, and that he knows who I am” (Benzies and Magill-Evans, 2015).

Another said:

“He knows his dad – it’s so precious” (Benzies and Magill-Evans, 2015).

#### 3.1.2. Parents get to know, interact, and challenge their child through learning about the infant’s bodily expressions, capacity, and development

Learning to observe infant behavior and becoming sensitive to their infants expressions and needs, helped promote parental sensitivity to the boundaries of the infants’ expressed capacity and competencies (Gravem et al., 2013; Benzies and Magill-Evans, 2015; Håkstad et al., 2016; Lee et al., 2019; Øberg et al., 2019; Baraldi et al., 2020; Van Den Hoogen et al., 2021). The parents’ personal experience with the intervention was the foundation for this sensitivity building. Involvement and administering the interventions in a sensitive manner, adjusting the intervention to the parents’ observation of their infant’s state and responses, fostered emergence of reciprocity in interaction (Benzies and Magill-Evans, 2015; Lee et al., 2019; Øberg et al., 2019) and alleviated parental fear of interacting with their child (Benzies and Magill-Evans, 2015; Håkstad et al., 2016; Øberg et al., 2019). Parental observation of their infant’s independent movements validated the infant’s competency for the parent (Øberg et al., 2019). Regardless of the timing of the intervention parents indicated that learning about the infant capacities and developmental changes, as well as understanding their direct interaction with and facilitation of their child’s active participation, was a turning point in their relationship with and perception of their child’s abilities (Benzies and Magill-Evans, 2015; Lee et al., 2019; Øberg et al., 2019; Baraldi et al., 2020). One mother that had administered the intervention during the infants stay in the NICU described how her perception of her infant changed from the infant being vulnerable and fragile to being more like a normal infant:

“From the physical therapist I learned that my little boy could cope with a lot and it was not dangerous to handle him… I felt that my baby was more like a real baby and not a doll made of glass” (Øberg et al., 2019).

Another mother who had administrated the intervention program at home explained:

“I think it gave me quite a lot, regarding that, eh, I learned to think in another way, it opened my eyes, I had an “Aha-moment,” for example things that you take for granted, you think he does not understand, but all of a sudden, “oops, he understands”” (Baraldi et al., 2020).

### 3.2. Building parent empowerment through mutual interactions with the child

#### 3.2.1. Active participation and learning are key to confidence and fulfillment in the parental role

The practical knowledge parents learned through therapeutic interventions contributed to reflections of their role as a parent (Benzies and Magill-Evans, 2015), and helped parents be more comfortable in parenting their infant (Lee et al., 2019; Edney and Mchugh, 2021; Van Den Hoogen et al., 2021). The analysis revealed that administering the intervention gave a liberating feeling of empowerment and an affirmation to their role as a parent (Kynø et al., 2013; Benzies and Magill-Evans, 2015; Håkstad et al., 2016; Lee et al., 2019; Øberg et al., 2019; Edney and Mchugh, 2021; Van Den Hoogen et al., 2021; Ochandorena-Acha et al., 2022). Parents felt empowered through being the one in charge, being responsible for the caring for their infant and being the one promoting infant development (Kynø et al., 2012; Håkstad et al., 2016; Øberg et al., 2019; Baraldi et al., 2020; Ochandorena-Acha et al., 2022). In the NICU, commencement of the intervention became a critical moment where the infant became their baby (Øberg et al., 2019). Later, taking an active part in the intervention contributed to the feeling of being more capable of handling and playing with their infant (Lee et al., 2019; Ochandorena-Acha et al., 2022). Involvement in the intervention was perceived as regaining control over their child’s destiny and helping their child develop (Edney and Mchugh, 2021). Being the provider made them feel complete as a parent (Lee et al., 2019; Øberg et al., 2019; Van Den Hoogen et al., 2021) and gave a touch of normalcy to the family (Håkstad et al., 2016). One mother highlighted the importance of being independent and taking responsibility for the infant. She put it this way:

“We did the care all by ourselves. It was our own process and very meaningful for us to feel complete as a parent” (Van Den Hoogen et al., 2021).

Another parent explained that being involved in therapy made her confident in her role as a mother who can facilitate her child’s development:

“It is kind of one of the few places where she, where you are challenging her in a way, and not just, not just nurse her, kind of. And it is a good way to, in a way, well get, or at least some help to approach her, I think… That you, yes it kind of gets a touch of normalcy” (Håkstad et al., 2016).

#### 3.2.2. Gaining knowledge together with their therapist faded initial fears and promoted self-efficacy and enthusiasm

The transition from the role of being a passive parent, observing and comforting their infant to actively taking part in the parent–child interaction was experienced as challenging. Parents expressed they initially were afraid of hurting their infant when introduced to the intervention activities (Gravem et al., 2009; Øberg et al., 2019; Edney and Mchugh, 2021; Ochandorena-Acha et al., 2022). However, initial concerns and stressors like feeling useless, insecure, and being afraid were overcome through supervision and support from the therapist (Gravem et al., 2009; Håkstad et al., 2016; Øberg et al., 2019; Edney and Mchugh, 2021; Ochandorena-Acha et al., 2022). Verbal encouragement (confirmation and compliments), and collaborative relationships were highlighted as important for alleviating parental stress and fear (Øberg et al., 2019; Baraldi et al., 2020; Edney and Mchugh, 2021). The reassurance and confidence they developed when handling their infant promoted parenting self-efficacy (Kynø et al., 2012; Håkstad et al., 2016; Øberg et al., 2019; Baraldi et al., 2020), made it easier for parents to accept new challenges, and provided a tool and security for improvising (Kynø et al., 2012; Håkstad et al., 2016). Although worries about disability and excitement about milestones went hand in hand, parents appreciated the opportunity for involvement in the intervention (Edney and Mchugh, 2021). However, parents expressed the need for the therapist to be skillful (Gravem et al., 2009; Håkstad et al., 2016), and for easy access to therapists (Edney and Mchugh, 2021). Limited access to the therapist reduced the parent’s sense of self-confidence (Edney and Mchugh, 2021). One mother explained:

“I think [not being able to access the therapist] may have… affected my confidence… so for me it was like, well how do I do this? Like, do I do it this way, or that way?… the nurses only know what’s wrote down” (Edney and Mchugh, 2021).

#### 3.2.3. Gaining knowledge makes parents feel competent and promotes autonomy in daily life decision-making

The analysis also revealed that parent administered interventions which engage the infant in the therapeutic activities, is associated with a feeling of greater parent autonomy and heightened control of their decision making (Gravem et al., 2009; Kynø et al., 2013; Benzies and Magill-Evans, 2015; Håkstad et al., 2016; Lee et al., 2019; Øberg et al., 2019; Van Den Hoogen et al., 2021; Ochandorena-Acha et al., 2022). The know-how they developed through learning about and doing the intervention, was appreciated and supported parents in their ability to select effective parenting skills in other situations, adjusting and harmonizing their activities during parent–infant interactions and play (Benzies and Magill-Evans, 2015; Håkstad et al., 2016; Lee et al., 2019; Øberg et al., 2019). Parents were thus able to transfer their knowledge to other contexts, by building on the experience-based, interactional knowledge gained during intervention (Gravem et al., 2009; Kynø et al., 2012; Benzies and Magill-Evans, 2015; Ochandorena-Acha et al., 2022). Through incorporated practical knowledge parents went from feeling powerless to becoming empowered and fully participating in care and decisions (Edney and Mchugh, 2021; Van Den Hoogen et al., 2021; Ochandorena-Acha et al., 2022). A mother outlined how the intervention contributed to her capacity to handle her infant:

“Now (when asking about the early intervention) I am sure that the way I stimulate him is the correct (…) so, we are trained parents. When we came back home, we were trained. We were confident to do things (…) very positive, very. I mean, our word is: learning” (Ochandorena-Acha et al., 2022).

### 3.3. Translation of the findings into patterns

Our findings, based on results from the 10 primary studies, comprise a broad range of empirical data. The two developed preliminary concepts provided a growing curiosity toward any mechanisms of the individual categories which may contribute to uniting or separating them. This work became the foundation for our further development and synthesis of the primary analysis. Our focus was especially drawn towards how the active role of the parent being the interventionist appeared as overarching the two concepts and their categories. The active role of the parent, being the one acting, was significant both for the parents’ relationship building with the prematurely born infant and for fulfillment of their parental role. This significance of being active was apparent across the early intervention programs and their heterogeneity (e.g., commencement of the intervention, inclusion criteria, hospital vs. home based, dosage, content, duration, and the profession of the interventionist).

The prominent features of successful and meaningful participation in early intervention programs were especially associated with the parents’ sense-making processes, the fostering of mutuality in the interaction between parent and infant, their development of confidence, and the ability for parents to see new possibilities for actions within themselves, with and in the child. Translating this, we arrive at two key patterns of our findings: (1) active learning and doing embodied interactive interventions build mutuality in the parent–child relationship and (2) building knowledge through interactions with their child together with a skillful therapist fades fear and enables parents to fulfill their role with a sense of autonomy, confidence, and enthusiasm. In the discussion, we elaborate these patterns and their impact on clinical practice.

## 4. Discussion

This qualitative systematic synthesis reveals how parents’ direct involvement in early interventions is associated with improved parent–infant relationships, parental empowerment, and sense making processes. Additionally, these opportunities improve parental motivation and engagement. We are not the first to demonstrate these areas in relation to early interventions. They are also highlighted in previous research emphasizing the importance of involving parents in early interventions with their infant born preterm citing the impact the parent–infant relationship has on developmental outcomes both for the parent and the child (Spittle and Treyvaud, 2016). However, a critical imperative of the present systematic synthesis are the mechanisms by which motivation, engagement and sense-making were fostered, something that is not highlighted in recent literature.

To understand and elaborate on the mechanisms, we take an enactive approach. This is a conceptual framework on embodied cognition and intersubjectivity highlighting how our actions and engagement with the world simultaneously comprise cognitive experiences, sensorimotor, and affective processes (Fuchs and De Jaegher, 2009). The close relationship between cognition, bodily actions and experiences and the surroundings constitutes an embodied cognitive system. For this synthesis, we apply selected enactive concepts, i.e., embodiment, autonomy, participatory sensemaking, and agency, to explain how bodily interaction and handling relates to parents’ know-how, motivation for active participation and adherence to early intervention programs.

### 4.1. Active learning and doing embodied interactive interventions build mutuality in the parent–child relationship

Parent–infant attachment relationships and cooperative dyadic pattern of interaction are challenged when the infant is born preterm (Evans et al., 2014). It is well documented that prematurely born infants’ behavior, activity state, and emotional expressions can be difficult to understand (Muller-Nix et al., 2004; Feldman, 2006). The subtle, sometimes hardly detectable ways in which the infant born preterm interacts with the world into which she has been born, often cause uncertainty in parents’ approach (Feldman, 2006). Child behavior and facial emotional expressions that are difficult to identify can therefore, like parental psychological distress, adversely affect the developing parent–infant relationship, placing the relationship between parents and children under pressure. However, our analysis revealed how the parents’ active progressive embodied learning was associated with parental perception of engagement in mutually satisfying dyadic parent–infant interactions, attachment, and awareness of activities suitable for the infant’s developmental stage. Apparently, appropriately learning by doing as part of early intervention foster parental meaning, and thereby becomes motivational for the parents to adhere to the intervention. Embodied learning during face-to-face-interaction may nurture transformation of both parents and children, giving them an opportunity to be directly involved in a coordinated and synchronized manner, which is a central feature for creation of meaning and positive developmental environments (Fuchs and De Jaegher, 2009; Ulvund, 2022).

Learning about neurobehavioral development through practical demonstrations and explanations appear as an important basis for establishing rapport (Håkstad et al., 2016; Øberg et al., 2019), but the synthesis demonstrates how the perception of further comprehensive parental attachment was primarily augmented by first-hand bodily experiences. Active parental involvement illuminates the resonance between meaningful engagement and the construction of parental identity (Laney et al., 2015). What the synthesis shows regarding the interactions between the parent and the child, provides insights into how their bodily processes involving cognitive and sensorimotor experiences, contribute to connecting the dyad through both expressions and the perceptions of expressive behavior of the other. During such interactions corresponding feelings, also known as mutual affective resonance (Fuchs and De Jaegher, 2009), can be evoked and gradually developed within the dyad. We hypothesize that both the parent and the infant may experience the feeling of affecting each other, enhancing emotions, mutuality, and synchronized rhythms in the parent–child interaction. The experience of affecting one another through the coupling of coordinated nonverbal behavior with coordinated physiological response among the dyad appear to be what enables the parent’s and child’s mutual affections, something that is considered a central contribution to the feeling of attachment (Feldman, 2015). Thus, through coordination of biological and social processes during social contact, biobehavioral synchrony (i.e., the mechanism by which attachment bonds become dyad specific and long lasting) can occur (Feldman, 2015), giving the idea of what promotes satisfaction and performance of the intervention. This understanding is supported by previous research that describe the critical component of attachments precisely to be the coordination of biological and behavioral processes between the parent and the child during social contact (Feldman, 2017). Thus, when implementing early intervention in infants born preterm, an important aspect for infant and parental development and parental engagement and adherence to early intervention, appears to include the parent–infant in a two-way process of perceiving and being perceived, acting, and being acted upon.

Parents interacting directly with the infant allows gaze, attitude, movements, and handling contribute to regulate the behaviors of both the parent and the infant and thereby enhancing the co-constitution of who parent and child are allowed to be and become for each other in the very situation, strengthening the bonding processes. According to the enactive approach, their direct experiences in the interaction processes arise from the individuals’ self-constituted bodies (De Jaegher, 2015). Even the infant born preterm will at birth have an inherent emerging form of agency on a biological level that is coordinated with evolving sensorimotor capacities, enabling her to adapt and respond to the environment (Quintero and De Jaegher, 2020; Sørvoll et al., 2022), including the parents. Accordingly, as self-producing and self-maintaining subjects, the parents’ and the child’s intentions are expressed in and through their bodies, making them able to perceive the intentions of the other, also described as participatory sense-making (De Jaegher, 2015). The infant’s needs and concerns can thus appear for the parent as the parent incorporate and develop their knowledge during direct interaction with their child. Our findings suggest that parents handling the infant while implementing the intervention, is what enhanced development of parental awareness and embodied sensitivity in perceiving the infant’s cues. Consequently, as highlighted in previous research (Nordhov et al., 2010; Ulvund, 2022), the infant’s ability to regulate her state and behavior can increase when facing a sensitive and attentive parent, which may foster her sensorimotor development.

In sum, this synthesis demonstrates how parents commonly experience relationships with their infant as meaningful, serving as an important aspect to develop understanding of the purpose of the intervention and to promote parental engagement and participation. For clarity then, it happens directly in interaction through sensemaking activities that the parent and child reciprocally move in their emotional and affective attachment (De Jaegher, 2015), being a means to generate parental motivation, engagement and meaning. Parental experience of significant closeness to their infant through mutually satisfying nurturing relationship, involves physical proximity (e.g., to hold the child in the lap) related to entering a reciprocally bodily interplay. Features like engagement, motivation, and willingness to adhere to early intervention can then be understood as rooted and fostered in experiences of what emerges through mutuality in interaction processes. Such interaction will override the parent’s individual feelings and intentions as the parent–infant relationship develop a momentum of its own. We suggest that the perception of mutuality in interaction also may trigger further motivation for generation of new developmentally appropriate activities as the infant grows and develops.

### 4.2. Building knowledge through interactions with their child together with a skillful therapist reduces fear and enables parents to fulfill their role with a sense of autonomy, confidence, and enthusiasm

Our analysis revealed how the parents’ active progressive embodied learning together with their infant not solely was significant for the developing parent–infant relationship, but also associated with guidance and a positive trustful collaboration with a skillful therapist. The importance of professional parent–infant social support has previously been acknowledged for parents’ feeling of confidence in caring for their infants born preterm (Gibbs et al., 2019; Harniess et al., 2022). These studies highlight the importance of professionals’ style of communication and collaborative relationships for parental confidence (Gibbs et al., 2019; Harniess et al., 2022). However, the richness of parental narratives in the original studies of this synthesis provide a recognition beyond social support demonstrating parents’ perceptions of becoming an empowered parent through a route of active involvement in early interventions including parent, infant and therapist. Several mechanisms to grow into the parental role are likely to be involved, including relational processes, co-creation of know-how and parental agency, beliefs in the intervention, and transformation of parental self.

This synthesis shows that an important consideration in establishing relational processes is the two-ways enabling relations between therapist and parent developed through professional competence and parental willingness to participate in the intervention. The core of professional competence in the original studies relates to both verbal and physical guidance. This illuminates that enabling two-ways relationship calls for therapists exercising professional competence in a manner that parents can relate to, i.e., parents perceiving the therapist as an individual to whom the infant matters like the infant do for them. In this context the therapist becomes a person the parent can connect with through affective feelings simultaneously as both parties are distinct from each other and have their own perspectives. Our findings therefore highlight the significance of the role of embodied interaction processes in establishing and transforming meaning to both parent and therapist. These participatory sense-making processes also allow know-how to be expressed and unfold for both parties (Fuchs and De Jaegher, 2009; De Jaegher et al., 2016). We suggest that the parents’ knowing evolves from their self-finding and self-correcting processes with the therapist in which meaning and understanding of the child’s expressions and needs are co-created, and parental know-how enhanced. Evolving affective feelings to the infant may also enhance parental self-confidence and beliefs in the therapist and in the intervention. Parental know-how is thus fostered by interactional and embodied processes in which the processes of embodied interacting itself play an important role for understanding, self-efficacy in handling the infant and thereby development of self-confidence (Fuchs and De Jaegher, 2009; Fantasia et al., 2014). Gradually with evolving parental know-how and self-confidence the parent–child interaction becomes more independent of the therapist. A feeling of control in daily interactions with the infant enable parents to act and interact with their surroundings in a more flexible way which relates sense of agency to parental autonomy providing the parents a feeling of control of own choices and actions.

Consequently, self-confidence may affect and enhance the parental identity (Laney et al., 2015). The findings demonstrate that taking part in the interventions by direct involvement with the infant, transformed the parents’ identity in relationship to becoming a parent as they became more confident in reinforcing nurturing infant interaction and play. We speculate that increased self-confidence provides the parents with a sense of agency to (inter)act and make qualified choices and decisions for the infant and themselves. It further appears that becoming a parent is a negotiation between an increasing sense of autonomy and role-related identity. In this regard parents appear to incorporate parenthood into their identities and who they perceive themselves in the role as a parent. Thus, based on the analysis and in line with a previous report of the influence of motherhood on women’s identity development (Laney et al., 2015), becoming a parent of an infant born preterm seem to be related to an expansion of the self, which includes parents incorporating their infants into their identities, self-possibilities, and boundaries. If so, parents will likely attend to issues and engage in meaningful interactions they perceive as relevant and significant regarding the infant and her wellbeing and development, while ignoring other things. The findings therefore point to how early intervention may be a means that can help parents achieve self-efficacy and thereby triggering motivation and enthusiasm through participatory sense-making. Motivation and enthusiasm then become a driving force that pushes the parent to participate and adhere to early intervention over time.

The analysis further shows that the above-mentioned processes are like a circuit where the various processes are co-connected. The core mechanism for participation and adherence occurs as participatory sensemaking through active parental involvement. For participatory sensemaking to happen the analysis points to availability of sufficient therapy resources with skilled therapists for parents when they need guidance to carry out the intervention. It becomes clear that features highlighted by the parents include the therapists’ know-how about infants born preterm, handling, and genuine interest in the child.

### 4.3. Methodological considerations

The methodological approach with specific criteria prior to the literature search, guidelines to classify the overall quality of the included papers, and involvement from several authors through the different analysis processes strengthen the rigor of this study. All five authors are pediatric physiotherapists with work experience from both primary and secondary healthcare settings and represent perspectives from a Norwegian and American physiotherapy and research context. Sharing a common interest in professional practices especially interactive processes in pediatric physiotherapy may have impacted preconceptions, choices, and interpretations. However, the authors represent different fields of training and hold various assumptions, experiences, and perspectives, which guided and enriched the research processes. Established assumptions and positions were questioned and challenged throughout the study and have thus improved the conceptual thinking and the rigor and quality of the research (Malterud, 2001).

Our analysis was based on results from 10 primary studies that comprised a broad range of empirical data from a total of 193 parents of infants born preterm in Canada, Norway, Netherlands, Sweden, Spain, Republic of Korea, United Kingdom, and United States. The studies exhibited heterogeneity regarding inclusion criteria and the focus, delivery dosage, onset and termination of the intervention and the follow-up protocols. In addition, the professionals involved in teaching the parents came from diverse professional backgrounds. Finally, the sample subjects are from different countries and cultures and participated in different models of early intervention, which can potentially influence the generalizability of the findings to other contexts. Despite these differences, many of the parents’ experiences were similar.

It should be noted, we are aware that although our systematic search was extensive, it was limited to publications written in Scandinavian and English languages. We recognize additional papers may be available in other languages, providing supplementary or alternative viewpoints. However, considering the studies included were performed in eight different countries providing the rich information included in this meta-synthesis, we concluded our sample has sufficient data for a meta-synthesis on the field of interest. In addition, a detailed and transparent description analysis was provided.

### 4.4. Final remarks

This systematic analysis demonstrates that ‘active embodied doing’ is the basis for facilitating sense-making, know-how, autonomy, parental becoming, and therefore, supports parental motivation for active participation and adherence to early intervention programs with infants born preterm. A particularly important mechanism for this motivation is the change in the parental perception of their infant, which evolved through the development of mutuality in the parent–infant relationship and reciprocity in the parent-interventionist cooperation during embodied interactions. Consequently, an important consideration when designing, planning, and implementing early intervention programs is to promote embodied parent–infant interactions and promoting trust between the parent and the interventionist.

## Author contributions

GKØ conceptualized and designed the qualitative systematic synthesis, supervised the literature search, was involved in the analysis, prepared the figure and tables, drafted, and revised the manuscript. MS was involved in the analysis, in drafting the initial manuscript, and reviewed the manuscript. CL performed the literature search, was involved in the analysis, drafted the figure and the tables, and reviewed the manuscript. GLG reviewed the manuscript for important intellectual content, revised, and proofread the manuscript. RBH performed the literature search, was involved in the analysis, and reviewed the manuscript. All authors contributed to the article and approved the submitted version.

## Conflict of interest

The authors declare this paper was conducted in the absence of any commercial or financial relationships that could be considered as a potential conflict of interest.

## Publisher’s note

All claims expressed in this article are solely those of the authors and do not necessarily represent those of their affiliated organizations, or those of the publisher, the editors and the reviewers. Any product that may be evaluated in this article, or claim that may be made by its manufacturer, is not guaranteed or endorsed by the publisher.
